# Health insurance and antibiotic prescription in pharyngitis: a cross-sectional study in Saudi Arabian primary healthcare centers

**DOI:** 10.3389/fmed.2026.1814507

**Published:** 2026-06-05

**Authors:** Aouab Abdul Khafez, Amro Abdel-Azeem, Malak Alotaibi, Rehab Almubrick, Hala Tamim, Naif Alotaibi, Noara Alhusseini

**Affiliations:** 1 Department of Biostatistics, Epidemiology and Public Health, College of Medicine, Alfaisal University, Riyadh, Saudi Arabia; 2 Population Health Management and Research, Riyadh First Health Cluster, Ministry of Health, Riyadh, Saudi Arabia; 3 School of Kinesiology and Health Science, York University, Toronto, ON, Canada; 4 Department of Surgery, College of Medicine, Alfaisal University, Riyadh, Saudi Arabia

**Keywords:** acute pharyngitis, antibiotic prescribing, antibiotic resistance, health insurance, prescription incentives

## Abstract

Inappropriate antibiotic prescribing for acute pharyngitis remains a key driver of antibiotic resistance and a persistent public health challenge. This study examined antibiotic prescribing patterns and their association with demographic characteristics and health insurance factors among acute pharyngitis visits in Saudi Arabian governmental primary healthcare centers. A cross-sectional analysis was conducted using anonymized electronic health records for 14,550 acute pharyngitis visits by Saudi nationals in 2023 within governmental primary healthcare centers. Descriptive statistics, chi-square tests, and multivariate logistic regression were used to assess predictors of antibiotic prescription, including age, gender, insurance scheme, and private insurance company. Antibiotics were prescribed in 84.7% of visits, with amoxicillin (62%) and azithromycin (37%) accounting for the majority of prescriptions. Younger age, particularly children aged 0–7 years, was significantly associated with higher odds of receiving antibiotics, while gender was not a significant predictor. Similarly, to age, the patient’s insurance scheme was a predictor of antibiotic prescription, with patients covered by governmental insurance being 28.6% more likely to receive antibiotics than those under private insurance. These findings indicate a high prevalence of antibiotic use for acute pharyngitis in primary care and suggest that prescribing practices are primarily influenced by patient age and insurance scheme, underscoring the need for strengthened antibiotic stewardship and improved adherence to clinical guidelines.

## 1 Introduction

Antibiotics are drugs that kill bacteria and stop their growth. They are seen as critical tools for preventing and treating infections in people, animals, and crops (1). The introduction of the term “antibiotics” was the result of successfully isolating chemical substances from microorganisms capable of inhibiting the growth of other microbes, done by the microbiologist Selman Waksman and his team. However, it was Alexander Fleming’s unanticipated identification of penicillin in 1928 that marked the initiation of modern antibiotic therapy (2).

The post-World War II period, often referred to as the “golden era” of antibiotics, is where the penicillin discovery team stumbled upon penicillinase, a bacterium capable of degrading the antibiotic penicillin, even before widespread access to the antibiotic (2). The use of penicillin in clinical therapy started in 1943, and a decade later, penicillin resistance had already become a major clinical problem (3).

Microorganisms such as bacteria and viruses develop resistance to antimicrobials by constantly evolving, rendering the antimicrobial useless, and earning themselves the name “superbugs” (4). This same occurrence could be seen with each new antibiotic (3). The mechanism of this resistance comes in multiple forms, including active antimicrobial efflux, biofilm formation, reduced drug entry into pathogens’ cells, altered drug targets, enzymatic metabolism of antimicrobial agents to inactive products, and protection of antimicrobial targets (5).

According to the World Health Organization, more than 700,000 lives worldwide are lost due to drug-resistant infections. It is estimated that by 2050, the number could exceed 20 million. With an expected financial loss of $2.9 trillion (4).

In 2019, antibiotic resistance killed more people than any other infectious disease. In 2020, the world faced one of the worst pandemics in history. Due to this pandemic, high-income countries reported increased antibiotic use, leading to increased antibiotic resistance. Simultaneously, articles from low- and middle-income countries show substantial increases in multidrug-resistant (MDR) infections during the COVID-19 pandemic. These unprecedented events have overwhelmed the healthcare system, pushing it toward excessive misuse of antibiotics. Reversing the progress achieved in the prior years (3).

Due to their high production costs and short shelf life, large pharmaceutical companies have opted out of developing antibiotics. Since 2017, only eight new antibacterial agents have been approved, and no new antibiotic classes have been added since the 1980s, further accelerating the inevitable (6).

Pharyngitis is inflammation of the oropharynx mucous membrane. The cause is usually viral, affecting approximately 50% to 80 % of cases, but it can also be bacterial, allergic, or caused by certain toxins. In 2010, pharyngitis accounted for 1.8 million emergency visits. 693,000 of which were under the age of 15 (7). Before 2015, antibiotics were prescribed for 49.3% of pharyngitis cases and at higher rates among adults (54.4%) than in pediatrics (45.0%) (8). Prior to the COVID-19 pandemic, the average monthly rate of antibiotic prescriptions for pharyngitis cases in urgent care was 38.9%. During the pandemic period, the amount rose to 50.6%, of which 70% were prescribed without any testing (9).

In Saudi Arabia, pharyngitis is the cause of more than half of the antibiotics given in primary care centers, along with Bronchitis and Tonsillitis. Yet only 10%–20% of pharyngitis cases in adults and 30% in children are attributable to bacterial causes (10). The Ministry of Health has established a Group A Streptococcal Pharyngitis Protocol built on the Modified Centor scoring system. This protocol was first developed to offer guidance on safe and cost-effective management of acute pharyngitis. Additionally, the guideline includes the antibiotics and doses to be used, based on the patient’s status, with clear emphasis on narrower-spectrum antibiotics such as “Penicillin V” (10).

The Constitution of Saudi Arabia states that the government provides public-sector healthcare services to all its citizens, free of charge, with no out-of-pocket contributions by beneficiaries (11). This is financed by the government, which allocates an average of 16.5% of its annual budget to the health sector (12) under the supervision, control, and management of the Ministry of Health (11).

4.28 million Saudi Arabians are covered by private health insurance (13). The Council of Health Insurance in Saudi Arabia mandates that all private-sector employers must provide health insurance coverage to their employees (14) and their dependents (15). Additionally, employees can purchase insurance policies with a broader network of healthcare providers, specifically in the private sector (16). However, treatment under private insurance coverage is not entirely free, as cost-sharing agreements are in place (17).

Cost-sharing is an out-of-pocket payment made by the consumer that can serve as a financial tool for the insurer to encourage beneficiaries to avoid unnecessary services and incentivize demand for valuable care, as the financial burden will be shared with the insured. Deductibles, copayments, and coinsurance are all forms of cost-sharing (18). The payment amount is set, and mentioned in the policy, and is only typically applied in outpatient settings, as emergencies and inpatient cases are excluded for the most part (17).

Understanding how insurance payment structures influence treatment decisions is essential. Higher fees, scheduled payments, and low denial rates by intermediaries incentivize providers to do more once a patient is through their door. An individual’s insurance plan could also affect a physician’s willingness to see the patient. Since insurance schemes, like Medicaid, historically paid physicians less than two-thirds of what private insurers pay for the same service (19). International evidence further demonstrates the effect. In India, insurance schemes were associated with increased consumption of healthcare services, with utilization rising by 244% among insured households compared to non-insured households. It also showed that the increase in healthcare utilization was for simpler conditions, such as fever. Most of the rise in use of services was in private facilities, with some states reporting that 100% of the medical claims were from private healthcare providers (20). Similar patterns can be seen elsewhere, as an insured individual is more likely to use medical services than the uninsured. However, differences arise among insurance schemes. Privately insured patients reported the highest propensity to use cost-effective routine medical care, whilst the publicly insured reported a higher tendency to use the least cost-effective mixture of healthcare services (21).

Financial barriers implemented in insurance schemes, such as co-payments, will also shape the patient’s behavior. Co-payments as low as $5 for prescriptions can lead to higher medical expenses. Patients of high socio-economic deprivation will not collect their medications regularly, or use the drugs less to last them longer, leading to an increased risk of hospitalization (22). And after the abolishment of prescription fees in Scotland, hospitalization for asthma and chronic obstructive pulmonary disease was remarkably decreased (23). However, these dynamics intersect with the concept of moral hazard, where individuals change their behavior and consumption of health services because of insurance coverage. The lower the price of care, the more the person is incentivized to consume it, which would lead to overconsumption and waste. Along with consumer moral hazard, provider moral hazard, also known as provider-induced demand, contributes heavily to the increase in healthcare costs (18).

There is growing recognition that prescribing practices are shaped not only by clinical judgement but also by the broader environment in which physicians operate. In many health systems, healthcare providers are regularly exposed to pharmaceutical promotion, with every $1 spent by a pharmaceutical company in marketing to doctors, it would yield $2.64, a return on investment of 164% (24). A survey conducted among 2,000 healthcare providers in the United States of America found that key experts presenting at sponsored conferences and industry-hosted events had a notable influence on their prescription behavior, with nearly two-thirds prescribing off-label medications (25).

Antibiotic prescribing for pharyngitis provides a clear example of how these external pressures interact with clinical practice. Evidence from Nigeria shows that over 75% of healthcare providers reported prescribing antibiotics to patients complaining of a sore throat. More than 60% prescribed antibiotics as a precaution. Notably, over half the physicians agreed that their behavior potentially promotes antimicrobial resistance (26). A similar trend in Riyadh, Saudi Arabia, was seen among physicians in 10 different teaching hospitals, showing that only 27% adhered to the pharyngitis antibiotic guidelines, mainly because signs and symptoms are sufficient to assess the case. An interesting rationale behind going against the guideline was the pressure from parents to prescribe antibiotics regardless of the lab result (27). Another local study reported that 80 % of pharyngitis cases in Saudi Arabia received antibiotics in primary care centers, although only 28% were evidence-based prescriptions. Of those prescriptions, 34% were first-line antibiotics, while the majority received broad-spectrum antibiotics (28).

Patterns of inappropriate antibiotic prescription have also been linked to specific patient and provider characteristics. Older male patients were more likely to receive unnecessary antibiotics. Whilst provider traits such as being in a rural area, having a high patient turnover rate, and being in the medical practice for a long time were linked with more inappropriate antibiotic prescription behavior (29). Even when employing all possible interventions, there is still a notable prevalence of inappropriate prescribing behavior. A commonly mentioned cause for opposing the guideline and prescribing unwarranted antibiotics was the patient’s expectation (30).

Misuse is further influenced by patient behaviors and knowledge gaps. A study measuring awareness levels in Saudi Arabia showed that 67.7% had poor knowledge and awareness regarding antibiotic resistance. The main cause of antibiotic use in participants was a sore throat at 83.3%, over one-third of the participants have taken antibiotics without a prescription, and 33.3% have used leftover antibiotics from previous illnesses (31).

## 2 Materials and methods

### 2.1 Study design

A cross-sectional study using secondary data from 2023 on acute pharyngitis cases documented by primary healthcare centers under the supervision of Health Holding, a state-owned company providing healthcare services through health clusters located in all regions of Saudi Arabia.

### 2.2 Study population and data source

The study consisted of 14,550 visits for acute pharyngitis by Saudi nationals in governmental primary healthcare centers managed by Health Holding in 2023. Data were collected from anonymized electronic health records extracted from the Riyadh First Health Cluster (R1), one of the 20 clusters under Health Holding. R1 provides healthcare services through 126 primary healthcare centers registered on the Raqeem system, a cloud-based digital health record system that integrates the Saudi health ecosystem. The data were filtered to include visit claims with ICD-10 codes for acute pharyngitis (J02-J02.8-J02.9).

The dataset included the following variables:

- Patient Age: Continuous variables / categorized (0–7, 8–17, 17–30, 31–40, >41).
- Patient Gender.
- Insurance Scheme.
- Insurance Company ID: Numerically coded upon extraction for confidentiality.
- Antibiotic Given Status.
- Antibiotic Name.

### 2.3 Inclusion criteria

- Claims with acute pharyngitis as a diagnosis in 2023.
- Claims documented with insurance details (governmental or private).
- Saudi Arabian citizens.

### 2.4 Exclusion criteria

- Claims without recorded insurance information.
- Claims with incomplete or missing prescription data.
- Non-Saudi residents.

### 2.5 Data management and processing

Data were cleaned and processed to ensure consistency and accuracy before analysis. Insurance company IDs were numerically coded at first, then later alphabetically coded and randomly assigned to mask the actual names for confidentiality. The reason for the change from numerical coding was to anonymize the data further and enhance the readability and interpretation of the results.

### 2.6 Ethical consideration

This study adhered to ethical guidelines for data privacy and patient confidentiality. All patient identifiers were removed, and data were analyzed in aggregate form. Approval was obtained from the King Saud Medical City institutional review board, reference number: B0RE-13-Jan25-01, prior to data extraction and analysis.

## 3 Results

### 3.1 Demographic characteristics

Table 1 presents the overall demographic characteristics of the study participants (*n* = 14,550). The mean age of the participants was 22.59 years with a standard deviation of 17.63 years, indicating a wide age range and considerable variability within the study sample. The gender distribution was nearly balanced, with females accounting for 49.1% of the participants and males 50.9%. With regard to insurance coverage, the majority of participants were enrolled in governmental insurance schemes (91.2%), while a smaller proportion (8.8%) was covered by private insurance.

**Table 1:** Demographic characteristics of the acute pharyngitis patients (*n* = 14,550).

Characteristic	Category	Count	%
Age	Mean (22.59)	—	—
	SD (17.63)	—	—
Gender	Female	7,146	49.1%
	Male	7,404	50.9%
Insurance scheme	Governmental	13,263	91.2%
	Private	1,287	8.8%
Total	14,550		

Table 2 further illustrates the distribution of participants’ age groups and gender by insurance scheme.

**Table 2:** Distribution of participants’ age and gender according to insurance scheme (*n* = 14,550).

Characteristic	Category	Governmental	Private
Age	0–7	3,358	179
	8–17	3,752	149
	18–30	1994	459
	31–40	1767	243
	≥41	2,392	257
Gender	Female	6,502	644
	Male	6,761	643
Total	14,550		

**Figure 1:**
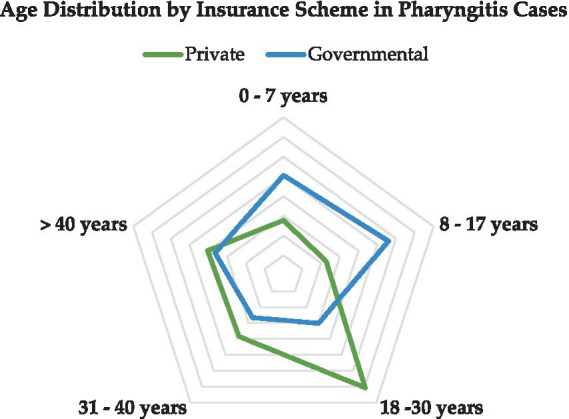
Age distribution by insurance scheme in pharyngitis cases.

In Figure 1, we can see the distribution of insurance scheme beneficiaries by age group. A significant proportion of private insurance beneficiaries are in the 18–30 years group (35.3%), followed by the >41 years group (20.2%). On the other hand, the majority of governmental insurance beneficiaries (53.3%) are in the 0–7 years and the 8–17 years age groups.

### 3.2 Antibiotic prescription

Table 3 presents the overall prevalence of antibiotic prescriptions among the study participants (*n* = 14,550). The majority of participants (12,331; 84.7%) received antibiotics, while 2,219 participants (15.3%) did not. This indicates a high overall rate of antibiotic prescription in the study population.

**Table 3:** Frequency and percentage of antibiotic given (n = 14,550).

Characteristic	Category	Count	%
Antibiotic given	Yes	12,331	84.7%
	No	2,219	15.3%

Table 4 further breaks down antibiotic prescription status by gender, age group, and insurance scheme. By gender, 85.1% of females and 84.4% of males received antibiotics, showing a fairly balanced distribution between males and females.

**Table 4:** Frequency and percentage of antibiotic prescription status according to gender, age, and insurance scheme (*n* = 14,550).

Characteristic	Category	Antibiotic Given	
		Count / (%)	
		Yes	No
Gender	Female	6,082 (85.1%)	1,064 (14.9%)
	Male	6,249 (84.4%)	1,155 (15.6%)
Age	0–7	3,078 (87.0%)	459 (13.0%)
	8–17	3,328 (85.3%)	573 (14.7%)
	18–30	2,029 (82.7%)	424 (17.3%)
	31–40	1,661 (82.6%)	349 (17.4%)
	≥41	2,235 (84.4%)	414 (15.6%)
Insurance Scheme	Governmental	11,288 (85.1%)	1,975 (14.9%)
	Private	1,043 (81.0%)	244 (19.0%)

Analysis by age group reveals that younger participants had slightly higher prescription rates. The 0–7 years group had the highest proportion receiving antibiotics (87.0%), followed by the 8–17 years group (85.3%). Adults aged 18–30 and 31–40 had slightly lower rates (82.7 and 82.6%, respectively), while participants aged ≥41 years had 84.4%.

Regarding the insurance scheme, governmental insurance participants had a higher rate of antibiotic prescriptions (85.1%) compared to those with private insurance (81.0%).

**Figure 2:**
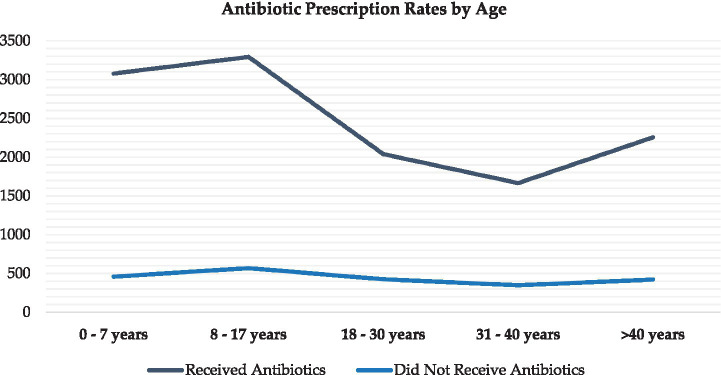
Antibiotic prescription rates by age.

Figure 2 illustrates the prescription of antibiotics by age group. The 0–7 years group had the highest percentage of antibiotics prescribed, while the 8–17 years group had the highest number of antibiotics prescribed. The prescription rate declined through the 18–30 years and the 31–40 years group, reaching the lowest point, before increasing again for the >41 years group. In contrast, the number of patients who did not receive antibiotics stayed relatively low across all age groups.

### 3.3 Antibiotic used

Table 5 presents the frequency of different antibiotic types prescribed to participants (*n* = 12,331). Amoxicillin was the most commonly prescribed antibiotic, given to 7,612 participants (62%), followed by Azithromycin with 4,624 participants (37%). Other antibiotics, such as Cephalosporin, Ciprofloxacin, Clarithromycin, and Sulfamethoxazole, were prescribed much less frequently (≤0.4%). All antibiotics used for acute pharyngitis visits were broad-spectrum.

**Table 5:** Frequency and percentage of the antibiotic prescribed (*n* = 12,331).

Characteristic	Category	Count	%
Antibiotic name	Amoxicillin	7,612	62%
	Azithromycin	4,624	37%
	Cephalosporin	50	0.4%
	Ciprofloxacin	11	0.1%
	Clarithromycin	5	0.04%
	Sulfamethoxazole	29	0.2%

### 3.4 The relationship between demographic variables and antibiotic dispensing

A chi-square test of independence was conducted to examine whether antibiotic dispensing differed significantly across key demographic characteristics, including insurance scheme, gender, and age group (Table 6).

**Table 6:** Chi-Square test: the relationship between demographic variables and antibiotic dispensing (*n* = 14,550).

Category	Value	df	Asymptotic Significance (2-sided)
Insurance scheme	15.019	1	
Gender	1.419	1	0.234
Age	30.186	4	<0.001

The analysis showed a significant association between insurance scheme and the likelihood of receiving an antibiotic prescription (*χ*^2^(1) = 15.019, *p* < 0.001), indicating that patients enrolled in different insurance schemes were not prescribed antibiotics at equal rates.

Similarly, age group demonstrated a strong and significant relationship with antibiotic dispensing (χ^2^(4) = 30.186, *p* < 0.001). This finding indicates that antibiotic prescribing patterns vary substantially across age categories, with certain age groups being more likely to receive antibiotics than others.

In contrast, gender did not show a statistically significant association with antibiotic dispensing (χ^2^(1) = 1.419, *p* = 0.234). This result suggests that male and female patients received antibiotics at comparable rates, and gender was not a determining factor in prescribing decisions within the sampled population.

Overall, the results indicate that insurance type and age play meaningful roles in shaping antibiotic prescribing patterns, whereas gender does not contribute significantly to variations in antibiotic use. These findings justify further multivariate modeling to determine whether these associations persist after controlling for additional factors.

### 3.5 Relationship between insurance company ID and antibiotic prescription behavior

A Chi-square analysis was performed to examine the association between private insurance company identity and antibiotic prescription behavior (Table 7). The analysis was limited to visits covered by private insurance schemes, resulting in a total of 1,287 valid cases.

**Table 7:** Chi-square test of the relationship between insurance company identity and antibiotic prescription (*n* = 1,287).

Test	Value	df	*p*-value (2-sided)
Pearson Chi-Square	27.366	22	0.198

The Pearson Chi-square test showed no statistically significant association between insurance company ID and antibiotic prescribing behavior among privately insured patients (χ^2^ = 27.366, df = 22, *p* = 0.198). This finding indicates that the likelihood of receiving an antibiotic prescription did not differ significantly across private insurance companies.

### 3.6 Antibiotic prescription based on age, gender, and insurance scheme

A logistic regression analysis was performed to determine which patient characteristics are associated with the likelihood of receiving an antibiotic prescription and to identify which groups are more exposed (Table 8).

**Table 8:** Binary logistic regression of antibiotic prescriptions based on age, gender, and insurance scheme (*n* = 14,550).

Variable	Category	OR (Exp(B))	Adjusted OR	95% CI Lower CI–Upper CI	*p*-value
Gender	Male	1.057	1.055	0.963–1.155	0.250
	Female (Ref)	—	—	—	—
Age group	0–7	1.242	1.234	1.068–1.425	0.004
	8–17	1.076	1.064	0.927–1.221	0.379
	18–30	0.886	0.913	0.786–1.061	0.235
	31–40	0.882	0.890	0.761–1.040	0.143
	≥41 (Ref)	—	—	—	—
Insurance scheme	Governmental	1.337	1.286	1.086–1.467	0.002
	Private (Ref)	—	—	—	—

Gender was not associated with antibiotic prescription. Males had similar odds of receiving antibiotics as females (OR = 1.057; AOR = 1.055, 95% CI: 0.963–1.155, *p* = 0.250), indicating no meaningful difference in prescribing patterns between the sexes.

Age showed a selective effect rather than a gradual trend. Only children aged 0–7 years had significantly higher odds of receiving antibiotics compared with the reference older age group (OR = 1.242; AOR = 1.234, 95% CI: 1.068–1.425, *p* = 0.004). The other age categories (8–17, 18–30, and 31–40 years) were not significantly different from the reference group (*p* > 0.05), suggesting that increased antibiotic use is concentrated in early childhood rather than across all younger ages.

The insurance scheme demonstrated a significant association with prescribing behavior. Patients covered by governmental insurance were more likely to receive antibiotics than those covered by private insurance (OR = 1.337; AOR = 1.286, 95% CI: 1.086–1.467, *p* = 0.002).

Overall, antibiotic prescription in the study population was influenced by insurance coverage and young age, while gender showed no detectable effect.

## 4 Discussion

The aim of this study was to examine the pattern of antibiotic prescriptions for the management of acute pharyngitis across both governmental and private insurance programs in Saudi Arabian primary health care settings, with specific reference to the role of various factors in antibiotic prescribing.

The study revealed that antibiotic prescribing for the management of the disease was high among the patient population studied, with the majority of patients receiving antibiotics (84.7%). This high prescription rate aligns with previous international studies (26), as well as studies done in Saudi Arabia (28). The study also shows that only broad-spectrum antibiotics were used, with no adherence to the Ministry of Health’s protocol advocating for a more conservative, first-line approach with narrower-spectrum antibiotics (10). However, low adherence to antibiotic guidelines by physicians is expected, as this study’s findings are consistent with other local studies (27, 28). This reflects a potential over-prescription and misuse issue that warrants further investigation and intervention.

At the bivariate level, the insurance scheme was found to be significantly associated with antibiotic dispensing. After adjusting for age and gender in the regression analysis, patients covered by governmental insurance were 28.6% more likely to receive antibiotics than those under private insurance. Aligning with international studies, where patient cost-sharing played a significant role in reducing medical services (22, 23). Yet, in contrast to others (19, 20), where the presence of insurance was associated with increased healthcare utilization. This suggests that the potential difference in prescription rates is not solely due to demographic characteristics but rather reflects system-level or practice-environment factors that influence prescription behavior.

Age was also identified as an important variable influencing antibiotic prescription. Chi-square tests demonstrated a strong relationship between age groups and antibiotic dispensing (*p* < 0.001). Regression analysis further showed that children aged 0–7 years were more likely to receive antibiotics than older patients, contrary to the literature (8, 29). This finding is expected, as young children often present with less specific symptoms, or, as local studies stated, parenteral pressure (27).

On the other hand, gender was found not to be related to antibiotic prescriptions, neither in bivariate nor in multivariate analysis. The rate of antibiotic prescriptions was similar for men and women, suggesting that prescribing decisions are independent of patient gender.

Similarly, differences in antibiotic prescriptions among private insurance companies were not statistically significant. This could be down to the relatively smaller sample size of private insurance patients, which limited the detection of meaningful differences.

## 5 Limitations

Several limitations should be considered.

1) The study relies on data from governmental primary healthcare centers only, potentially limiting the generalizability to private healthcare providers, as the difference in settings could prompt different clinical behavior.
2) The imbalance within the population under study: Governmental insurance among participants was as high as 91%. This would make comparisons of results across insurance groups difficult and limit generalizability to private insurance beneficiaries.
3) Physicians may not always be aware of a patient’s insurance status during clinical encounters, as noted in operational practices. Clinical covariates, such as comorbidities, disease severity, and lab results, were unavailable. Therefore, we were unable to determine whether the antibiotic was prescribed correctly for each visit.
4) This study analyzed visit data, meaning each sample represents a visit, not an individual, and does not account for correlations from repeated visits by the same patient, as personal identifiers were removed prior to data extraction.
5) Potential clustering and geographic effects across different healthcare centers were not assessed because center IDs were anonymized to preserve privacy.

## 6 Conclusion

Antibiotic prescription for acute pharyngitis in Saudi Arabian primary healthcare settings is remarkably high, with the solitary use of broad-spectrum agents and limited adherence to the MOH treatment protocol.

Age emerged as a key determinant of antibiotic prescription, while gender was not associated with altered prescribing behavior. Highlighting the need for targeted stewardship at the pediatric level.

After adjusting for age and gender, the insurance scheme appeared to be the most significant predictor of antibiotic prescription, suggesting that government-insured individuals had higher odds of receiving antibiotics. However, the absence of significant variation in prescriptions among private insurance company beneficiaries leads us to suggest that insurance status and type alone might not have played a major role in influencing prescription behavior. Regardless, more research on the topic is needed.

## Data Availability

The original contributions presented in the study are included in the article/supplementary material, further inquiries can be directed to the corresponding author.
